# False Discovery Rate Control in Cancer Biomarker Selection Using Knockoffs

**DOI:** 10.3390/cancers11060744

**Published:** 2019-05-29

**Authors:** Arlina Shen, Han Fu, Kevin He, Hui Jiang

**Affiliations:** 1 The Blake School, 511 Kenwood Pkwy, Minneapolis, MN 55403, USA; ahshen20@blakeschool.org; 2 Department of Biostatistics, University of Michigan, 1415 Washington Heights, Ann Arbor, MI 48109, USA; fu.607@osu.edu (H.F.); kevinhe@umich.edu (K.H.)

**Keywords:** cancer biomarker, diseases genes, variable selection, false discovery rate, knockoffs

## Abstract

The discovery of biomarkers that are informative for cancer risk assessment, diagnosis, prognosis and treatment predictions is crucial. Recent advances in high-throughput genomics make it plausible to select biomarkers from the vast number of human genes in an unbiased manner. Yet, control of false discoveries is challenging given the large number of genes versus the relatively small number of patients in a typical cancer study. To ensure that most of the discoveries are true, we employ a knockoff procedure to control false discoveries. Our method is general and flexible, accommodating arbitrary covariate distributions, linear and nonlinear associations, and survival models. In simulations, our method compares favorably to the alternatives; its utility of identifying important genes in real clinical applications is demonstrated by the identification of seven genes associated with Breslow thickness in skin cutaneous melanoma patients.

## 1. Introduction

The discovery of biomarkers that are informative for cancer risk assessment, diagnosis, prognosis and treatment predictions is crucial. Many biomarkers have been proven to be very informative for clinical usage, with prominent examples such as BRCA1 and HER2 in breast cancer [1,2], EGFR in non-small-cell lung carcinoma [3] and PSA in prostate cancer [4]. Recent advances in high-throughput genomics make it plausible to select biomarkers from the vast number of human genes in an unbiased manner. For instance, genes associated with disease-related clinical outcomes can be identified by linking a patient’s gene expression to the disease progression [5] or other disease phenotypes. Furthermore, by understanding the regulatory roles of these associated genes on various cancers, treatment strategies may be developed. For these reasons, many gene signatures have been discovered for a variety of cancers.

However, many challenges exist for the selection of genes from the high-throughput and high-dimensional expression data at a genomic scale. Besides computational challenges due to the large size of data, a critical statistical difficulty is the control of false discoveries of all identified genes mainly due to the large number of genes versus the relatively small number of patients in a typical cancer study. The conventional method for genomic data analysis is known as univariate analysis, that is, exploring the relationship of the disease-related outcomes with one gene at a time. Due to its simplicity and intuitiveness, univariate analysis has been widely used in gene selection. However, high correlations exist among genes induced by co-expression activities, and hence genes correlated with disease-related genes are also correlated with disease outcomes (a.k.a. spurious correlation). Therefore they will be selected via univariate analysis, leading to high false discoveries. Another issue of univariate analysis is its low statistical power of identifying any disease-related genes due to the multiplicity of hypothesis testing [6] as well as noise that is unaccounted for. That is, relatively fewer genes that are truly associated with the outcome will be identified from univariate analysis than that with multivariate analysis. For the reasons above, penalized multivariate analysis approaches such as the lasso regression [7] and its extensions such as penalized generalized linear models and the Cox proportional hazard model with elastic-net penalty [8,9] have been applied recently to genomic data analysis [10,11]. Nevertheless, because cross-valuation is typically used for the selection of the optimal tuning parameters, such approaches often fail to control false discoveries [12]. This aspect has been clearly illustrated in our simulations in Section 3.1.

For prediction purposes, genes with spurious correlations to the disease outcomes may be useful. However, they are unsuitable when the goal is to understand the disease etiology, or to identify potential treatment targets, where genes that are genuinely associated with the disease are required. In other words, when the number of false discoveries is high, the discoveries are not scientifically replicable. Due to the high cost to experimentally validate the selected genes, there is an urgent need to control for false discoveries in gene selection procedures. The false discovery rate (FDR) [13], defined as the expected proportion of false discoveries among all discoveries, is a widely used method to control for false discoveries in genomic studies, due to its high statistical power compared with conventional methods that control for family-wise error rates (FWER) such as the Bonferroni correction. Controlling for FDR leads to limited proportion of non-true findings among all findings produced by a given analysis and discovery procedure, which translates to reliable scientific discoveries as well as reduced attempts and costs to validate non-true findings. The importance of controlling for the false discovery rate in lasso regression has also been recognized. Recently, [12] proposed a bootstrap/resampling method to control the FDR in lasso type variable selection. The smoothness of the limiting distributions of the bootstrap, which is the standard assumption for the bootstrap, is needed for such methods [14]. In [15], a knockoff procedure was introduced to control the FDR in linear regression when the number of variables is not too large; knockoff variables are constructed to mimic the correlation structure found within the existing variables. In a follow-up paper [16], the method was further expanded to a general framework and a high-dimensional situation for Gaussian variables was studied extensively. However, there is still a gap between the generally simple knockoff framework and the complicated data structures in real world applications.

In this paper, we propose several novel strategies based on the knockoff framework for variable selection subject to control for the false discovery rate. The proposed method is general and flexible, accommodating arbitrary covariate distributions, linear and nonlinear associations, and survival models. Simulation experiments and a real data example on gene identification for Breslow thickness in skin cutaneous melanoma patients demonstrate the utility of the proposed method.

## 2. Methodology

In many practical situations, identification of a set of explanatory variables which are truly associated with the response is a primary interest in investigation. This is particularly true in biomedical research when genes are selected from a pool of candidate genes that are potentially associated with a disease. To assure that most of the discoveries are true and replicable, one must know whether the false discovery rate, or the expected fraction of false discoveries among all discoveries, as defined in Definition 1, is acceptable or too large. In other words, the false discovery rate in this discovery process needs to be controlled at a desirable level.

**Definition 1** (False discovery)**.** *Let S be the true set of variables associated with an outcome, and $\hat{S}$ be the set of variables selected based on a dataset. The false discovery proportion (FDP) is defined as the proportion of false discoveries among all discoveries, i.e., $FDP:=|\hat{S}\backslash S|/|\hat{S}|$, where |·| is the size of a set, with the convention 0/0=0. The false discovery rate (FDR) [13] is defined as the expectation of FDP, i.e., FDR:=E[FDP].*

The method proposed in this paper is based on the knockoff framework first proposed in [15] and later generalized in [16]. The knockoff framework provides a recipe for building algorithms to control for FDR in variable selection. Under certain mild conditions, the FDR can be theoretically guaranteed to be controlled at a pre-specified level. The key contribution of the knockoff framework is the introduction of the concept of knockoff variables, as defined in Definition 2.

**Definition 2** (Knockoff variables)**.** *A set of random variables $\left({\tilde{X}}_{1},\cdots ,{\tilde{X}}_{p}\right)$ is said to be model-free knockoffs [16] for $\left(X_{1},\cdots ,X_{p}\right)$ with respect to response Y if they are constructed without looking at Y, and for any j∈{1,⋯,p}, the pair $\left(X_{j},{\tilde{X}}_{j}\right)$ is exchangeable conditioned on all the other variables $\left({\tilde{X}}_{1},\cdots {\tilde{X}}_{p}\right)$ and $\left(X_{1},\cdots ,X_{p}\right)$ excluding $\left(X_{j},{\tilde{X}}_{j}\right)$.*

In layman’s terms, each knockoff variable ${\tilde{X}}_{j}$ can be considered as a “fake” duplicate of the corresponding variable $X_{j}$, in that the relationship between ${\tilde{X}}_{j}$ and all the other variables and their knockoffs excluding $X_{j}$ is indistinguishable from the relationship between $X_{j}$ and all the other variables and their knockoffs excluding ${\tilde{X}}_{j}$. Furthermore, the knockoff variables are constructed without using the outcome variable, and therefore are guaranteed not to be associated with the outcome. As a result, in a variable selection procedure, a knockoff variable ${\tilde{X}}_{j}$ has equal chance of being selected as the “original” variable $X_{j}$ when $X_{j}$ is not associated with the outcome, which makes the knockoff variables robust benchmarks for FDR control. In this paper, we propose several novel strategies based on the knockoff framework for variable selection subject to control for the false discovery rate.

### 2.1. Construction of Model-Free Knockoff Variables

The first step for variable selection based on the knockoff framework is to construct knockoff variables. In [15,16], algorithms for constructing knockoff variables for low and high dimensional multivariate Gaussian distributions were proposed, respectively. In particular, an approximated algorithm was proposed in [16] to construct knockoffs by sampling from a multivariate Gaussian distribution with the same first two moments as that of the original variables. When the joint distribution of the original variables is known, the conditional distributions can be derived, based on which random samples can be drawn directly and can be used as knockoffs.

Although built on a multivariate Gaussian distribution, the performance of the knockoff variables constructed using the algorithm in [16] is reported to be quite robust against deviations from the Gaussian assumption, as long as the first two moments are approximated well. We also have the same observations in our experiments (See Appendix C). Therefore, we use the algorithm in [16] for the construction of knockoff variables for all the simulated and real data experiments in this paper, unless otherwise noted. Moreover, we propose another algorithm for constructing knockoff variables without the Gaussian assumption with much higher computational burden (See Appendix A), which may be used in situations when the Gaussian assumption is severely violated.

### 2.2. Model-Free Statistics

The knockoff framework guarantees that the FDR is controlled at a desirable level for variable selection. However, the statistical power for variable selection depends on the specific statistic being used in the knockoff framework. In [16], the lasso coefficient difference (LCD) statistic was proposed and shown to be very powerful for variable selection based on the lasso regression model. However, it assumes a linear relationship between the response variable and the predictors. When such relationship does not hold, the statistical power will be compromised. In this section, we propose two novel statistics to accommodate arbitrary relationships between the response and predictor variables, thereby realizing our goal of model-free variable selection. In contrast to the lasso regression model in [16], we incorporate machine learning techniques, such as support vector regression [17] and boosting [18], to allow for more flexible and complex model settings.

#### 2.2.1. Difference in R-Squared (DRS) Statistic

Intuitively, variable importance can be measured by the amount of variability of the response data explained by each specific variable. In practice, we can define a statistic named difference in R-squared (DRS) based on the difference between the $R^{2}$ value achieved by the full model and that by a partial model where one predictor variable is excluded at a time. See Appendix B for details.

#### 2.2.2. Risk Reduction in Boosting (RRB) Statistic

This statistic stems from the mboost R package which implements a functional gradient descent algorithm for model-based boosting. This method uses component-wise least squares estimates or regression trees as base-learners to optimize general risk functions. The algorithm is quite flexible in that it allows for various kinds of base-learners to be used, for example, linear, P-spline, and tree based base-learners, as well as a variety of loss functions and corresponding risk functions to be optimized. In a fitted boosting model, the accumulated in-bag risk reductions per boosting step for each base-learner or variable can be used to reflect variable importance. The amount of risk reduction can be provided by a function called varimp in the mboost R package with appealing computing efficiency. Similar to DRS, the risk reduction in boosting (RRB) statistic $W_{j}$ can be constructed by the difference between the risk reduction of variable $X_{j}$ and that of its corresponding knockoff ${\tilde{X}}_{j}$. Again, $W_{j}$ here attains the anti-symmetry property and a symmetric distribution under the null hypothesis. The high flexibility of the boosting method allows us to model arbitrarily complex relationships between *y* and $\left(X,\tilde{X}\right)$. The computational efficiency also makes this statistic favorable for our high-dimensional variable selection purpose. In our simulations, compared with the DRS statistic, we found that the RRB statistic achieves better performance in terms of FDR control and of statistical power for variable selection (See Appendix C), with much lower computational burden. Therefore, we use the RRB statistic for all the simulated and real data experiments in this paper, unless otherwise noted.

### 2.3. Nonlinear Screening

As genomic datasets are often high-dimensional, that is, the number of genes *p* is much larger than the sample size *n*, computing the statistics $W_{j}$ for each variable $X_{j}$ will take a lot of time. Here, we propose a nonlinear screening strategy to accelerate this procedure. In particular, when 2p>n, we perform univariate fitting of *y* to each $X_{j}$ as well as ${\tilde{X}}_{j}$, using nonlinear regression based on B-splines. In particular, we rank all the variables and their knockoffs based on the $L_{2}$ norm of the block-wise gradient vector. The top variables are corresponding to the steepest descent directions, which minimizes the direction derivative, and hence, provides the largest decrease in the linear approximation of the objective function. We then retain the top *n* variables for computing their $W_{j}$’s subsequently using a chosen statistic, and set the $W_{j}$’s for all the remaining 2p−n variables to be zero. In our simulations, we found that this nonlinear screening strategy can substantially reduce computational time while maintaining the FDR control as well as statistical power for variable selection (See Appendix C). Therefore, we use this nonlinear screening strategy for all the simulated and real data experiments in this paper, unless otherwise noted.

## 3. Results

### 3.1. Simulations

We first use simulation studies to evaluate the performance of our proposed method against two other existing methods: the knockoff method with lasso coefficient difference (LCD) [16] and lasso regression [7] with cross-validation (CV), a widely used variable selection approach. In simulations, we examine several situations to demonstrate that the proposed method performs well in terms of FDR control with increased statistical power. These simulations support the usage of the proposed method for analyzing a real dataset in Section 3.2. All simulations are performed in R.

In particular, we consider three cases of linear and nonlinear associations as well as survival models. In each case, we apply our proposal of using the boosting method with P-spline base-learners to approximate linear or nonlinear associations. We use the knockoff construction algorithm introduced in [16], the RRB statistic described in Section 2.2.2, and the nonlinear screening described in Section 2.3. Specifically, we use the mboost R package to fit *y* against the augmented design matrix $\left(X,\tilde{X}\right)$. For fitting lasso penalized models in the knockoff with the LCD method of [16] and in lasso regression with cross-validation, we use the glmnet R package [8,9] with five-fold cross-validation for selection of the regularization parameter of lasso in simulations for linear (Section 3.1.1) and nonlinear (Section 3.1.2) associations and the Cox proportional hazards regression [19] in simulations for survival analysis (Section 3.1.3).

#### 3.1.1. Linear Associations

**Figure 1:**
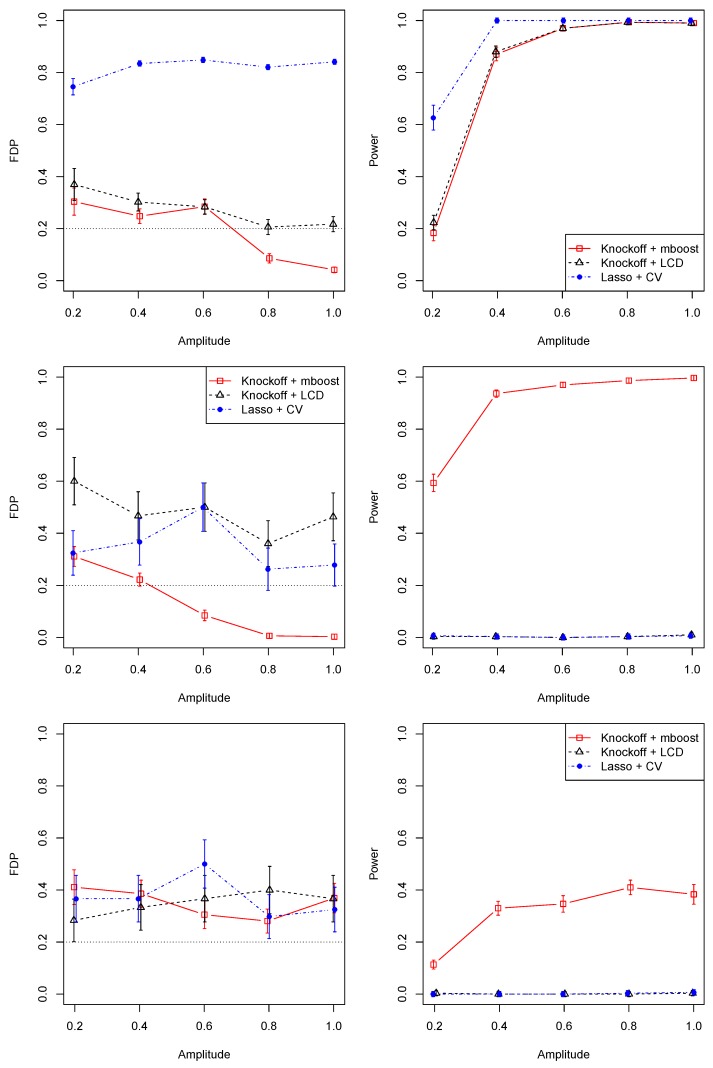
Simulation results for linear associations (**top panel**), nonlinear associations (**middle panel**) and survival analysis (**bottom panel**). Left panel: averaged false discovery proportion (FDP, the empirical version of FDR) and the standard error bars for knockoff variable selection with mboost (red), lasso coefficient difference (LCD) (black) and lasso regression with cross-validation (CV) (blue) as a function of amplitude (association strength (e.g., correlation) between a biomarker and the outcome) based on 30 simulation replications. The reference lines indicate the target false discovery rate of 20%. Right panel: corresponding empirical statistical power of the three methods.

The first simulation study focuses on linear associations in regression. In particular, the data were simulated from a linear regression model

(1) $$Y=\sum_{j=1}^{p}X_{j}\beta_{j}+\varepsilon ,\;\varepsilon ∼N\left(0,\sigma^{2}\right),$$

in which $X={\left(X_{1},\cdots ,X_{p}\right)}^{T}$ is distributed according to a *p*-dimensional Gaussian distribution N(0,Σ), with the ij-th element of Σ being $\rho^{\left|i-j\right|}$, following an auto-regressive variance structure with the auto-regressive coefficient ρ. Moreover, X and ε are independent. Of *p* variables, we randomly choose *k* variables $X_{j_{1}},\cdots ,X_{j_{k}}$ and set the corresponding $\beta_{j_{l}}=\zeta_{j_{l}}A$, where *A*, called amplitude, is a varying magnitude given in Figure 1, $\zeta_{j_{l}}$ is a random sign, and $\beta_{j}=0$ if $j\notin \{j_{1},\ldots ,j_{k}\}$. The amplitude represents the association strength (e.g., correlation) between a biomarker and the outcome. In this case, we simulate p=2000, k=10, ρ=0.3, and $\sigma^{2}=1$ from (1) with sample size n=300. This mimics the real data analysis in Section 3.2. We use the multivariate Gaussian distribution for its simplicity in simulating correlated covariates and the fact that the knockoff framework is robust against deviations from this distributional assumption, as long as the first two moments are approximated well [16]. Furthermore, the relationship between outcome and covariates can be arbitrary.

As suggested by Figure 1, the FDR is controlled around our target value of 20% for the proposed method (knockoff + mboost). The FDR for the knockoff + LCD method is slightly higher. In contrast, the FDR of the lasso + CV method is so high that the discovery is unreliable. All three methods have similar statistical power, and power increases and gets close to 1 as the signal strength gets stronger. A statistical power of 1 means the ideal situation that all genes that are truly associated with the outcome are identified. Although Lasso + CV has the highest power, it is not desirable for discovery, given the uncontrollable FDR levels. Thus, lasso + CV is not a suitable approach for gene selection.

As will be seen in the cases of nonlinear associations (Section 3.1.2) and survival models (Section 3.1.3), the proposed method becomes more powerful when the model assumption of linear associations is violated.

#### 3.1.2. Nonlinear Associations

Our second simulation study deals with nonlinear relationships in regression, in which we again compare the proposed knockoff + mboost method with the knockoff + LCD method of [16] as well as lasso + CV. Here, we replace $\sum_{j=1}^{p}X_{j}\beta_{j}$ in (1) by $\sum_{j=1}^{p}X_{j}^{2}\beta_{j}$ to accommodate nonlinear associations. All other settings are the same as in Section 3.1.1.

As indicated in Figure 1, the FDR for the proposed method (knock + mboost) is controlled under the target value of 20%, as marked by the horizontal dotted line, whereas the FDRs for the other two methods are above the target level. In terms of statistical power, the proposed method is much better than the other two methods, which assume a linear predictor while the proposed method is more flexible without such assumptions.

#### 3.1.3. Survival Analysis

Our third simulation study concerns the Cox proportional hazards regression [19] with a nonlinear predictor $\sum_{j=1}^{p}X_{j}^{2}\beta_{j}$ as in Section 3.1.2. Specifically, we generate *y* from the Cox model with a baseline hazard rate equals to 0.002 and a hazard rate of censoring equals to 0.004. The event time follows a Weibull distribution with the shape parameter equals to 1 and scale parameter equals to the baseline hazard rate multiplied by the exponential of the predictor, i.e., $\exp(\sum_{j=1}^{p}X_{j}^{2}\beta_{j})$. The censoring time is also sampled from a Weibull distribution with the shape parameter equals to 1 and scale parameter equals to the hazard rate of censoring. The actual observation time is the smaller value between the event and censoring times.

As shown in Figure 1, all three methods roughly achieve the objective of controlling the FDR at the desired level of 20% with slight inflation. The proposed method exhibits much higher power than the other two as was the case in Section 3.1.2.

Based on the simulation studies, we conclude that the proposed method performs well for linear and nonlinear associations as well as survival models. In practice, we do not need to assume linear or non-linear association between the biomarkers and the outcome, and our method will identify biomarkers with high statistical power and well controlled FDR regardless of the type of association that is present in the dataset.

### 3.2. Cancer Data

In this section we apply our proposed method as described in Section 3.1 to a real dataset from a cancer study for the identification of genes that are associated with clinical outcomes. We investigate a skin cutaneous melanoma (SKCM) dataset, which contains the expression levels of 20,531 genes from 355 melanoma patients measured by RNA-Seq. The dataset is a part of The Cancer Genome Atlas (TCGA) project and publicly available from the TCGA data portal at https://portal.gdc.cancer.gov/. The aim is to identify a set of genes associated with the clinical variable of interest, called Breslow thickness.

Due to the large number of genes and the relatively small sample size, to expedite computation while enhancing the accuracy of identification, we apply a filtering rule to select genes whose mean expression levels exceed 1 normalized transcripts per million (TPM) and the *q*-value (corrected using the BH procedure [13]) from univariate correlation tests with the response less than 0.2. This leaves us 4171 genes to which to apply our method with the log-transformed Breslow thickness as the response. The predictor variables are measured in log-transformed gene expression values (in TPM).

In this case, at a target FDR of 20%, our method identifies seven genes BOLA1 (BolA Family Member 1), CLDN16 (Claudin 16), EBF2 (EBF Transcription Factor 2), KCTD16 (Potassium Channel Tetramerization Domain Containing 16), KRT14 (Keratin 14), LOC100240735 (Uncharacterized LOC100240735), and MAP4K4 (Mitogen-Activated Protein Kinase 4). In the literature, the CLDN (Claudin) gene family is known to be associated with tumor suppressor genes; for example, hypermethylation of the CLDN11 promoter occurs frequently in malignant melanoma of the skin [20], which may encode a novel melanoma-specific tumor suppressor gene [21]. CLDN16 has been found to be associated with breast [22], thyroid [23], ovarian [24] and lung [25] cancers. Our finding suggests that CLDN16 is also associated with cutaneous melanoma of the skin, which seems consistent with the role of CLDN in terms of tumor suppression. Moreover, MAP4k4 belongs to the mammalian STE20/MAP4K family, which is often overexpressed in many types of human cancer and cancer cell lines, including malignant melanoma [26], because of its crucial role in transformation, invasiveness, adhesion, and cell migration [27]. KRT14 has been found to be associated with melanoma [28]. EBF2 has been found to be associated with prostate [29], bone [30], hematological and epithelial [31] cancers. KCTD16 has been found to be associated with thyroid cancer [32], while KCTD12, a member of the KCTD family, has been found to be associated with uveal melanoma [33]. BOLA1 and LOC100240735 (an RNA gene) are not known to be associated with any malignancies. To further understand the roles of these genes in melanoma, experimental follow-up studies are needed.

As a comparison, we also run Lasso + CV on the same dataset, for which a total of 140 genes are identified. Five of the seven genes identified by Knockoff + mboost are also identified by Lasso + CV. The two genes not identified by Lasso + CV are KRT14 and LOC100240735. Given the high false discovery rates of Lasso + CV in simulations (top-left panel of Figure 1), we expect a large proportion of these 140 genes to be false positives.

Furthermore, to demonstrate the performance of our approach in non-Gaussian data, we randomly pick 500 genes and assign 10 random genes among them to be truly associated genes with the remaining 490 genes to be null genes. We then randomly assign coefficients for the 10 truly associated genes by sampling from Uniform(1,5) with a random sign. To make the problem even more challenging and to demonstrate the ability of our approach working with non-quantitative data, we dichotomize the resulting linear predictor $Y=\sum_{j=1}^{p}X_{j}\beta_{j}$ at the median of its distribution so that the outcomes are binary (i.e., two groups of equal sizes). After running Knockoff + mboost at a target FDR level of 20%, a total of seven genes are identified, with five true positives and two false positives, which corresponds to an FDP of 28.6% and a statistical power of 50%.

## 4. Discussion

An advantage of our method is that no prior specification of the type of association (i.e., linear or non-linear) is needed, which is usually unknown for a given dataset. The knockoff construction algorithm in [16] is based on Gaussian assumption. Nevertheless, it seems robust for non-Gaussian data in our experiments. We also present a knockoff construction algorithm which does not require the Gaussian assumption in case such assumption is severely violated.

The statistical power depends both on the statistic being used and the correlation structure among covariates, which was also noted in [16]. As the correlation among covariates increases, the statistical power decreases. Therefore, a future research direction may be developing methods for the detection of highly correlated gene clusters that are associated with the outcome of interest. Furthermore, due to the high computational cost of building the knockoff variables, right now we can only practically use our method with up to around 5000 pre-selected genes. Thus, developing more efficient computational algorithms for building knockoff variables may be another future research direction.

The datasets and R programs for producing the results in this paper are available at http://www-personal.umich.edu/~jianghui/knockoff/.

## 5. Conclusions

The results in this paper demonstrate that our proposed approach can provide reliable false discovery rate control for variable selection in various statistical models. Such rigorous false discovery rate control is crucial for improving replicability of the findings and avoiding wasting resources for attempts to validate false discoveries. With additional enhancements, our method offers a promising avenue to identify reliable gene markers in cancer studies.

## Figures and Tables

**Figure A1:**
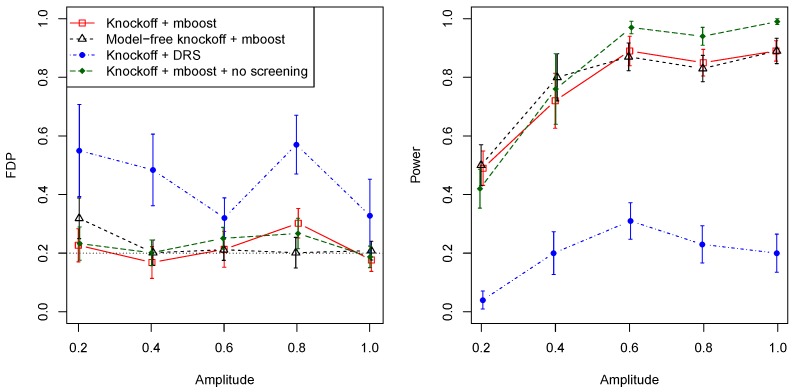
Simulation results for linear associations with log-normal covariates. Left panel: averaged false discovery proportion (FDP, the empirical version of FDR) and the standard error bars for knockoff variable selection with Knockoff with mboost (red), Model-free knockoff with mboost (black), Knockoff with DRS (blue) and Knockoff with mboost without screening (dark green) as a function of amplitude (association strength (e.g., correlation) between a biomarker and the outcome) based on 10 simulation replications. The reference lines indicate the target false discovery rate of 20%. Right panel: corresponding empirical statistical power of the four methods.

## Abbreviations

The following abbreviations are used in this manuscript:

FDR	False Discovery Rate
FWER	Family-Wise Error Rate
LCD	Lasso Coefficient Difference
SKCM	Skin Cutaneous Melanoma
TCGA	The Cancer Genome Atlas
TPM	Transcripts Per Million

## Appendix A. New Algorithm for Model-Free Knockoff Variable Construction

We propose a new algorithm for constructing knockoff variables without Gaussian assumption, by obtaining the conditional distributions empirically through regression models, regardless of the joint distribution of the covariates. Knockoff construction is independent from the response and the form of associations between response and covariates. Our proposal is to generate random samples from the conditional distributions by simply permuting the residuals, assuming that the residuals are approximately independently and identically distributed. Details of the algorithm are summarized in Algorithm A.1.

**Algorithm** **A.1** (Algorithm for construction of model-free knockoff variables)**.** For each covariate $X_{j}$, j=1,⋯,p,

(1) Fit $X_{j}$ on $\left(X_{-j},{\tilde{X}}_{1:j-1}\right)$ with a regression model, where $X_{-j}$ denotes $\left(X_{1},\cdots ,X_{j-1},X_{j+1},\cdots ,X_{p}\right)$ and ${\tilde{X}}_{1:j-1}$ represents existing knockoffs. No knockoffs are taken into consideration for $X_{1}$.
(2) Compute residuals $\varepsilon =(\varepsilon_{1},\cdots ,\varepsilon_{n})$ by subtracting the predicted value for $X_{j}$ from the corresponding observed values, i.e., $\varepsilon_{l}=X_{jl}-{\hat{X}}_{jl},l=1,\ldots ,n$, where ${\hat{X}}_{jl}$ is the predicted value of $X_{jl}$ by the regression model.
(3) Permute the residuals randomly, denoted by the permuted residuals $\varepsilon^{*}=\left(\varepsilon_{1}^{*},\cdots ,\varepsilon_{n}^{*}\right)$.
(4) Construct knockoff variable ${\tilde{X}}_{j}$ by adding the corresponding permuted residual to the predicted value for $X_{j}$, i.e., ${\tilde{X}}_{jl}={\hat{X}}_{jl}+\varepsilon_{l}^{*},l=1,\ldots ,n$.
(5) Proceed to the next covariate until all knockoffs are constructed.

Unlike [15,16], our proposed algorithm does not assume the multivariate Gaussian joint distributions of the covariates. The only requirement is the independence of the residuals, which may require an appropriate choice of regression model for fitting. For example, lasso [7] would be a good choice when $X_{j}$ is linearly dependent on $\left(X_{-j},{\tilde{X}}_{1:j-1}\right)$, and supervised machine learning techniques like support vector regression [17] and gradient boosting [18] are flexible enough to approximate nonlinear functional dependence. To avoid the problem of over-fitting, we may use *K*-fold cross-validation on test sets for prediction in subsequent calculations. Cross-validation may also help select optimal tuning parameters in the regression model and thus enable the method to be well adaptive to the observed covariate data.

Given the construction algorithm, we can generate knockoff variables from an arbitrary distribution, thus, effectively increasing the level of flexibility on the covariate distribution. For instance, for a binary response, we can simply replace the aforementioned regression models by classification models and then permute the binary response within the same prediction group to generate random samples for knockoffs.

The drawback of Algorithm A.1 is an increased computational burden. In our simulations, we noticed that the moments-based knockoff construction algorithm proposed in [16] is not very sensitive to the multivariate Gaussian assumption, and achieve similar performance as our regression-based knockoff construction algorithm in most cases (See Appendix C). Therefore, to save computing times, we use the algorithm in [16] for all the simulations and real data experiments, unless otherwise noted. Nevertheless, our regression-based knockoff construction algorithm has the potential to be used in broader scenarios, including situations when the Gaussian assumption is severely violated.

## Appendix B. Difference in R-Squared (DRS) Statistic

Algorithm B.1 gives the complete procedure for calculating the DRS statistics.

**Algorithm** **B.1** (The DRS algorithm)**.**

(1) Fit *y* with $\left(X,\tilde{X}\right)$ using a prediction model and obtain $R^{2}$, where $X=(X_{i},\ldots ,X_{p})$ contains the original predictor variables and $\tilde{X}=\left({\tilde{X}}_{1},\ldots ,{\tilde{X}}_{p}\right)$ contains the corresponding knockoff variables. $\left(X,\tilde{X}\right)$ is considered as an augmented design matrix with 2p columns.
(2) For each variable or knockoff variable in $(X,\tilde{X}),j=1,\ldots ,2p$, fit *y* with $\left(X,\tilde{X}\right)$ excluding the *j*-th variable with the same prediction model as in step 1) and obtain $R_{j}^{2}$. Calculate the absolute value of the difference between the two R-squared values, $Z_{j}=\left|R^{2}-R_{j}^{2}\right|,j=1,\ldots ,2p$, and record it as the importance score for the *j*-th variable (or knockoff variable).
(3) For j=1,…,p, the DRS statistic for $X_{j}$ can be derived as $W_{j}=Z_{j}-Z_{j+p}$, that is, the difference in *Z* between a variable and its knockoff.

The anti-symmetry requirement for feature statistics in [16] is fulfilled by the way we construct the DRS statistic. A large positive value of $W_{j}$ provides evidence that variable $X_{j}$ is strongly associated with the response *y*, while the statistic for a null variable is equally likely to take on a small positive or negative value, i.e., to have a symmetric distribution around zero. Similar to Algorithm A.1, we can apply various prediction methods for fitting in steps (1) and (2), for example, lasso for the linear relationship between *y* and $\left(X,\tilde{X}\right)$, and supervised machine learning techniques such as support vector regression and gradient boosting for nonlinear associations. To avoid the problem of over-fitting, we can use *K*-fold cross-validation and summarize the predictive power of the models by mean squared prediction error which can produce a cross-validated $R^{2}$. Cross-validation can also help select the tuning parameters in the prediction model and thereby enable the method to be well adaptive to the observed data.

## Appendix C. Additional Simulations

We conduct additional simulation experiments to compare four approaches: (1) knockoff construction using Gaussian based algorithm in [16] with RRB statistics in Section 2.2.2 (named Knockoff + mboost), (2) knockoff construction using model-free algorithm in Appendix A with RRB statistics (named Model-free knockoff + mboost), (3) knockoff construction using Gaussian based algorithm with DRS statistics in Appendix B (named Knockoff + DRS), and (4) knockoff construction using Gaussian based algorithm with RRB statistics but without nonlinear screening in Section 2.3 (named Knockoff + mboost + no screening). The simulation setting is similar to that of Section 3.1.1, except that here we exponentiate each element of the design matrix *X*, so that the covariates follow multivariate log-normal distribution. Furthermore, to save computing time, we let n=100 and p=100. The comparison results are shown in Figure A1. We can see that except for Knockoff + DRS which has an inflated FDP and a lower power, all three other methods have similar performance.
